# Supplementary material for: Extracting particle size distribution from laser speckle with a physics-enhanced autocorrelation-based estimator (PEACE)
Source: Nat Commun. 2023 Mar 1;14:1159. doi: 10.1038/s41467-023-36816-2 (PMC9977959; doi:10.1038/s41467-023-36816-2)
Supplement: Supplementary file 1 — Supplementary Information [file 41467_2023_36816_MOESM1_ESM.pdf]

From Laser Speckle to Particle Size Distribution in drying powders: A Physics Enhanced AutoCorrelation-based Estimator (PEACE)

Supplementary Material

Author list

Qihang Zhang[1], Janaka C Gamekkanda[2], Ajinkya Pandit[2], Wenlong Tang[3], Charles Papageorgiou[4], Chris Mitchell[4], Yihui Yang[4], Michael Schwaerzler[5], Tolutola Oyetunde[5], Richard D Braatz[2], Allan S Myerson[2], and George Barbastathis[6,7]*

1. Department of Electrical Engineering and Computer Science, Massachusetts Institute of Technology, Cambridge, Massachusetts 02139, USA.

2. Department of Chemical Engineering, Massachusetts Institute of Technology, Cambridge, Massachusetts 02139, USA.

3.Data Sciences Institutes, Takeda Pharmaceuticals International Co, 650 E Kendall St, Cambridge, Massachusetts 02142, USA.

4. Process Chemistry Development, Takeda Pharmaceuticals International Co, 40 Landsdowne St, Cambridge, Massachusetts 02139, USA.

5. Innovation and Technology Sciences, Takeda Pharmaceutical Company Limited, 200 Shire Way, Lexington, MA 02421

6. Department of Mechanical Engineering, Massachusetts Institute of Technology, Cambridge, Massachusetts 02139, USA.

7. Singapore-MIT Alliance for Research and Technology (SMART) Centre, 1 Create Way, Singapore 117543, Singapore.

* Email: gbarb@mit.edu

1. Apparatus overview.

Filter dryer overview. The filter drying device shown in Fig. S1 was designed based on a prototype[1]. The device has 1400 ml capacity with overall device dimensions of 330 mm height × 220 mm width × 220 mm length without the optical components. The body, impeller, and filter mesh are made of steel, and the lid is made of aluminum. The lid contains a vacuum port, an air/gas input port, a camera port, two wash solvent ports, a feed suspension port, and a glass observation window. A vacuum gauge and pressure release valve are attached to the lid. The impeller is connected to a motor on the top of the lid. The impeller blade is designed with teeth to promote cake depumping. An inductive heater and a thermocouple are attached to the bottom of the device to control the drying temperature. This inductive heating plate is used to heat the steel base and the steel body of the dryer. An endoscope camera is attached to the lid to record process

videos of the drying material from the top. The optics bench sheds light on the sample and collects the scattered beam through the observation window.

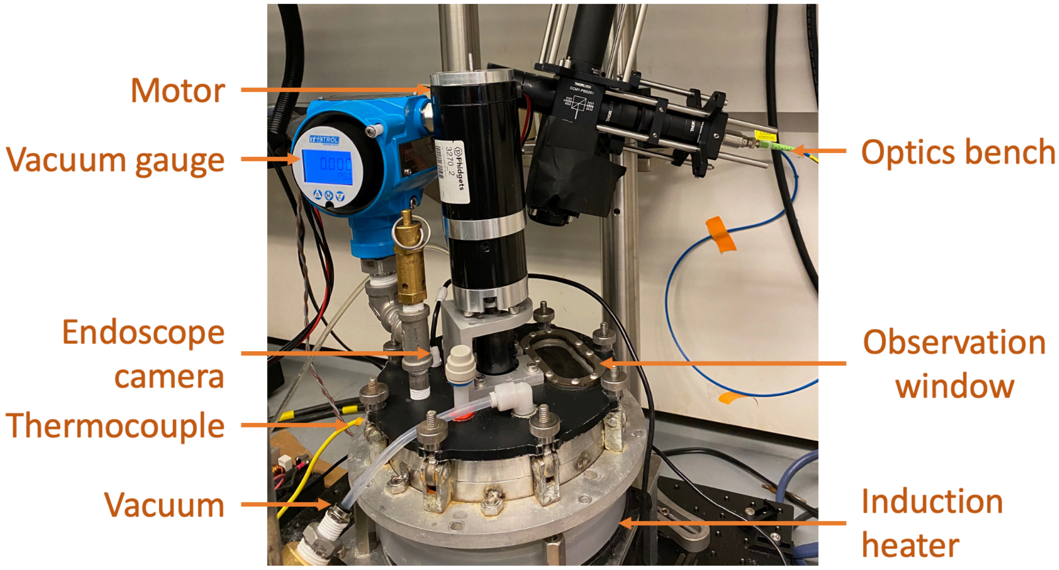

Fig. S1 | The photo of our drying filter. The crucial parts are labeled.

Optics bench. The laser model is Excelsior 532 Single Mode with 300 mW output power. After the fiber coupling, only 230 mW power injects into our system. Our lenses are made of N-BK7 with anti-reflection coating, whose transmission is 99.5% at 532 nm. The beam has a polarization ratio higher than 100:1. The extinction ratio of the polarized beam splitter (PBS) is higher than 1000:1. The transmission of the quarter plate and the silica window are 99.8% and 90%, respectively. The beam power on the sample surface is 203 mW. The filter dryer contains 150g KCl powder with a 690 J/kg·K specific heat capacity. From this calculation, our laser induces a 2.4 mK/s temperature rising rate, which is low enough not to disturb the drying process for the duration of observation. The power of the scattered light measured at the CCD plane is 120 µW. The coherence length of the laser is 25 m, corresponding to a temporal bandwidth of 0.01 pm. It provide sufficient temporal coherence to produce sharp speckles.

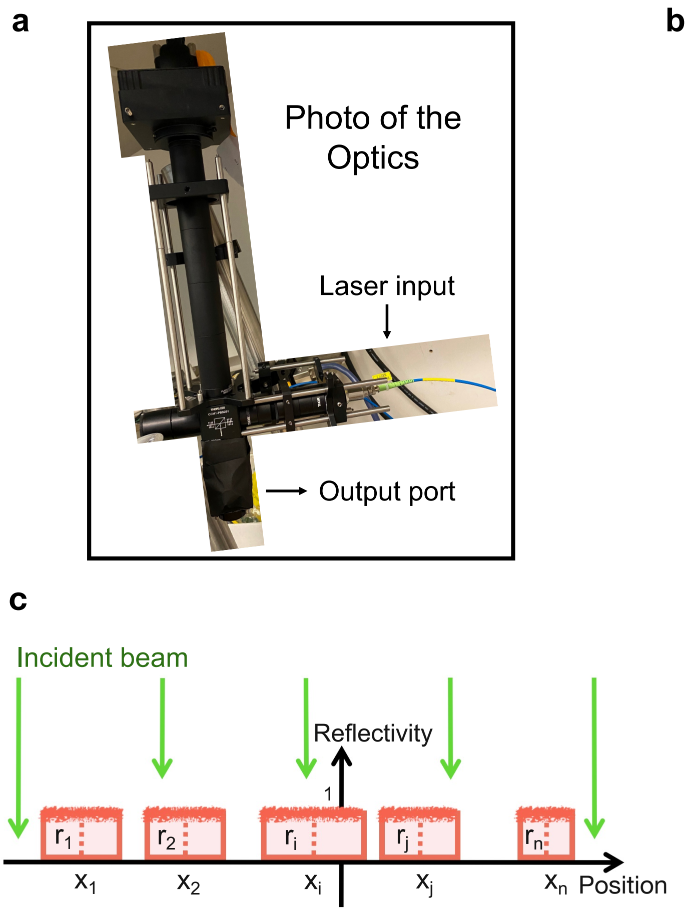

Fig. S2 | (a) A photo of our optics bench in the lab. All optical components are encapsulated in the optical tube and cubic to prevent light from escaping through the side. **(b)** A sketch of the optics. Lenses L1, L2, and L3 have focal lengths of 25 mm, 30 mm, and 250 mm, respectively. **(c)** A powder model sketch used in the derivation of the forward model. We only consider the top surface. The red rectangles are 1D powder particles, with radius r_i and position x_i. The rough top edge denotes that the scattered beam has a random phase at each different position.

Image acquisition. Our monochromatic CCD model is ZWO ASI183MM Pro with 5496 × 3672 pixels with a 2.4 μm pixel size. We run it in the bin-pixel mode with 1920 × 1080 pixels and 70 fps framerate. The typical size of the speckle pattern calculated from $\lambda f/D$ is 28 μm for our imaging system, which is larger than the pixel size to ensure a good resolution of the speckle pattern. We crop the central uniformly illuminated area to 1024 × 1024 pixels. Fig. S3 shows the single-frame raw speckle images and the corresponding autocorrelations with different exposure times. We find that 100 μs and 200 μs exposure times maintain a high degree of spatial correlation, whereas the speckle begins to decorrelate at exposures in excess of 500 μs. At exposure as high as 1 ms, the speckle pattern blurs and the corresponding

autocorrelation disperses. We choose 100μs as our exposure time to ensure that it is short enough to maintain the speckle spatial correlation within each frame. The time scale of PSD evolution in the powder drying dynamics is tens of minutes (as shown in Fig. 5) which is much longer than the time of a single PSD measurement. So we can safely assume that the powder PSD does not change during the data collection time.

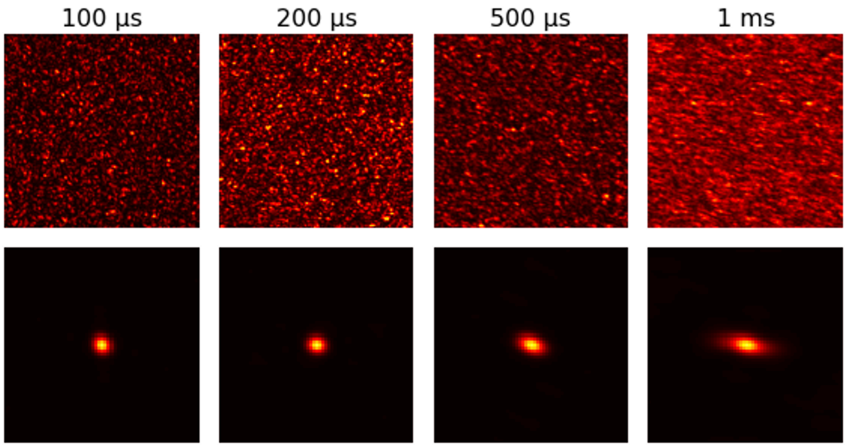

Fig. S3 | Speckle images (top) and its autocorrelations (bottom) in different exposure times.

2. Sample preparation and calibration.

Commercially obtained potassium chloride (KCl) powder was used to calibrate the speckle. The morphology of the KCl is blocked-shaped crystals. Twenty samples of KCl having different size distributions were prepared by sieving bulk KCl using sieves attached to the sieve shaker. The sieves were stacked in the sieve shaker in the order of decreasing sieve opening size from top to bottom (Sieve opening sizes used: 500 μm, 425 μm, 355 μm, 300 μm, 250 μm, 180 μm, 106 μm). The sieving was carried out 15-30 minutes until no further change of weight of the sieves with powder was observed. The samples obtained from the sieves were directly used to calibrate the speckle. Each sieved dry powder sample was added individually to the filter-dryer and stirring was carried out at 4 rpm to record 1000 frames speckle pattern. 20 calibration data sets were obtained corresponding to the sieved KCl samples.

From 1000 collected images in each sample set, we use 200 frames on average with a 40-frame step sliding window to obtain 20 averaged images. Employing an ergodicity argument, these averaged images are the ensemble averaged autocorrelations. To evaluate the generalization ability of our DNN model, we separate 10 sample sets to form the generator G training dataset, whereas the remaining 10 sets are the test dataset which is disjoint from the training process. Both datasets are kept away from the estimator F training process.

Offline particle size analysis was carried out by laser diffraction to obtain the ground truth particle size distribution of each KCl sample set. Malvern Mastersizer 2000 attached to a Scirocco 2000 dry dispersion unit was used to obtain particle size data. Fig. S4a shows the PSD data obtained from Mastersizer corresponding to each sieved sample. The finite sieves and master-sizer measurement limit the number of datasets we can collect, as they are time-consuming and unrecyclable.

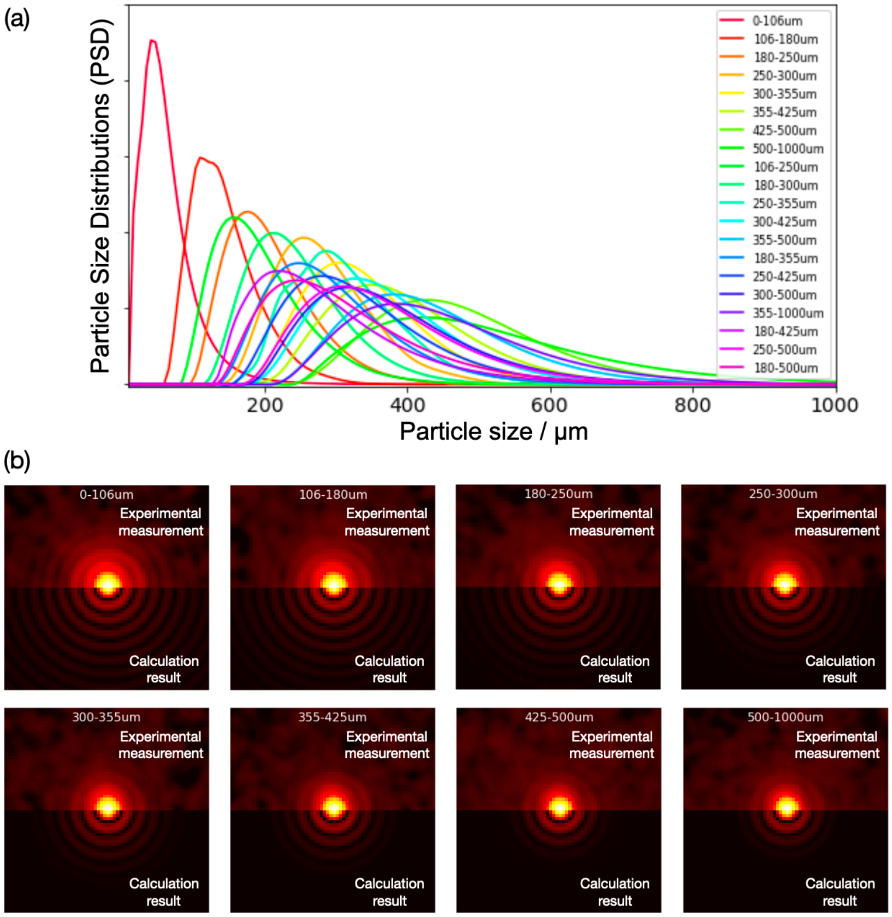

Fig. S4 | Sample preparation and calibration. (a) PSD calibration curves measured from the Mastersizer serve as the ground truth for each data set. **(b)** Measured average-autocorrelations and the calculations for 8 sample sets with ascending size distributions are plotted together. Their corresponding line-cut plots are shown in Fig.2(g).

3. Forward model.

The detailed math derivation of our forward model is described in this section. It continues from equation (4) in the Methods section. Substituting equations (2) and (3) into equation (4),

$$A(u') = \int \iint \iint e^{j\frac{2\pi}{\lambda f_3}[x(\xi_1-\xi_2)+(x+u')(\eta_1-\eta_2)]} S(\xi_1)S^*(\xi_2)S(\eta_1)S^*(\eta_2)dxd\xi_1 d\xi_2 d\eta_1 d\eta_2 \qquad (M1)$$

After integrating over x,

$$\begin{aligned} A(u') &= \iint \iint e^{j\frac{2\pi}{\lambda f_3}u'(\eta_1-\eta_2)} S(\xi_1)S^*(\xi_2)S(\eta_1)S^*(\eta_2)d\xi_1 d\xi_2 d\eta_1 d\eta_2 \int e^{j\frac{2\pi}{\lambda f_3}(\xi_1-\xi_2+\eta_1-\eta_2)x}dx \\ &= \iint \iint e^{j\frac{2\pi}{\lambda f_3}u'(\eta_1-\eta_2)} S(\xi_1)S^*(\xi_2)S(\eta_1)S^*(\eta_2)\,\delta(\xi_1-\xi_2+\eta_1-\eta_2)d\xi_1 d\xi_2 d\eta_1 d\eta_2 \qquad (M2) \end{aligned}$$

The delta function sifts the content of the integrand at $\xi_2 = \xi_1 + \eta_1 - \eta_2$. Then the equation can be further simplified as,

$$A(u') = \iint \iint e^{j\frac{2\pi}{\lambda f_3}u'(\eta_1-\eta_2)} S(\xi_1)S^*(\xi_1+\eta_1-\eta_2)S(\eta_1)S^*(\eta_2)d\xi_1 d\eta_1 d\eta_2 \qquad (M3)$$

From Equation (M3), we carry out variable substitution as $\eta = \eta_2$, $\sigma = \eta_1 - \eta_2$ and $\tau = \xi_1 - \eta_2$.

$$\begin{aligned} A(u') &= \iint \iint e^{j\frac{2\pi}{\lambda f_3}u'(\sigma+\eta-\eta)} S(\eta+\tau)S^*(\sigma+\eta+\tau)S(\eta+\sigma)S^*(\eta)d\tau d\sigma d\eta \\ &= \int d\tau \left[\int e^{j\frac{2\pi}{\lambda f_3}u'(\sigma+\eta)} S^*(\sigma+\eta+\tau)S(\sigma+\eta)d\sigma\right]\left[\int e^{-j\frac{2\pi}{\lambda f_3}u'\eta} S(\eta+\tau)S^*(\eta)d\eta\right] \\ &= \int d\tau \left[\int e^{j\frac{2\pi}{\lambda f_3}u'\sigma} S^*(\sigma+\tau)S(\sigma)d\sigma\right]\left[\int e^{-j\frac{2\pi}{\lambda f_3}u'\eta} S(\eta+\tau)S^*(\eta)d\eta\right] \\ &= \int d\tau \left|\int e^{j\frac{2\pi}{\lambda f_3}u'\sigma} S(\sigma)S^*(\sigma+\tau)d\sigma\right|^2 \end{aligned}$$

$$(M4)$$

Let $u = \frac{2\pi u'}{\lambda f_3}$,

$$A(u) = \int d\tau \left|\int e^{ju\sigma} S(\sigma)S^*(\sigma+\tau)d\sigma\right|^2 \qquad (M5)$$

After substituting S from equation (2),

$$A(u) = \int d\tau \left|\int e^{ju\sigma} a(\sigma)a^*(\sigma+\tau)w(\sigma)w^*(\sigma+\tau)d\sigma\right|^2 \qquad (M6)$$

In the internal integral over σ, the phase term $w(\sigma) = \exp\left(j\frac{2\pi}{\lambda}H(\sigma)\right)$ varies much faster than $a(\sigma)$nd $e^{ju\sigma}$, so we can apply the rotating wave approximation to move $w(\sigma)w^*(\sigma+\tau)$ outside the integral over σ.

$$A(u) = \int d\tau\, W(\tau) \left| \int e^{ju\sigma} a(\sigma) a^*(\sigma+\tau) d\sigma \right|^2, \qquad (M7)$$

where $W(\tau) = \langle w(\sigma)w^*(\sigma+\tau)\rangle_\sigma$ is the spatial average of $w(\sigma)w^*(\sigma+\tau)$ over σ. $W(\tau)$ describes spatial correlation of the surface phase. $W(\tau)$ will drop to 0 if τ is larger than the correlation length. For the ideal rough surface, the correlation length is infinitely small and $W(\tau)$ degrades into $\delta(\tau)$.

$a(x)$ is the mask defined by the particles, and we may express it as,

$$a(\sigma) = \sum_i Rect\left(\frac{\sigma - x_i}{r_i}\right) \qquad (M8)$$

$$a(\sigma)a^*(\sigma+\tau) = \sum_{i,j} Rect\left(\frac{\sigma - x_i}{r_i}\right) Rect\left(\frac{\sigma - (x_j - \tau)}{r_j}\right) \qquad (M9)$$

Where $\mathrm{Rect}(x) = 1 \text{ when } x \in [-1,1], \text{otherwise} = 0$ is the boxcar function. Since the particles cannot overlap, and the correlation length is well smaller than the particle size, we can approximately assume $\mathrm{Rect}\left(\frac{\sigma - x_i}{r_i}\right)\mathrm{Rect}\left(\frac{\sigma-(x_j-\tau)}{r_j}\right) = 0$ for $i \neq j$.

$$a(\sigma)a^*(\sigma+\tau) = \sum_i \mathrm{Rect}\left(\frac{\sigma - x_i}{r_i}\right)\mathrm{Rect}\left(\frac{\sigma - (x_i - \tau)}{r_i}\right) = \sum_i \mathrm{Rect}\left(\frac{\sigma - (x_i + \frac{\tau}{2})}{r_i - \frac{\tau}{2}}\right) \qquad (M10)$$

After substituting equation (M10) to equation (M7), we obtain

$$A(u) = \int d\tau\, W(\tau) \left| \sum_i \int e^{ju\sigma} \mathrm{Rect}\left(\frac{\sigma - (x_i + \frac{\tau}{2})}{r_i - \frac{\tau}{2}}\right) d\sigma \right|^2 \qquad (M11)$$

The internal integral is the Fourier transform

$$\int e^{ju\sigma} \mathrm{Rect}\left(\frac{\sigma - (x_i + \frac{\tau}{2})}{r_i - \frac{\tau}{2}}\right) d\sigma = e^{-ju\left(x_i + \frac{\tau}{2}\right)} \frac{\sin\left(u\left(r_i - \frac{\tau}{2}\right)\right)}{u}. \qquad (M12)$$

Substituting, we find

$$A(u) = \int d\tau\, W(\tau) \left| \sum_i e^{-ju\left(x_i + \frac{\tau}{2}\right)} \frac{\sin\left(u\left(r_i - \frac{\tau}{2}\right)\right)}{u} \right|^2 . \qquad (M13)$$

If we apply the assumption that $W(\tau) = \delta(\tau)$, then equation (M13) may be transformed as

$$A(u,t) = \left|\sum_i \frac{\sin(r_i u)}{u} e^{j2\pi x_i(t) u}\right|^2 . \qquad (M14)$$

Here, $u = \frac{u'}{\lambda f_3}$, i is the index of the i-th particle, and r_i and x_i are the radius and the position for the i-th particle, respectively. This is the same as equation (5) in the main text.

Below are the derivations of the ensemble average of the autocorrelation $A(u,t)$. The ensemble average is over independent measurements. Specifically, it is an average over x_i. However, x_i is a function of time t in our system since the powder are agitated by the impeller. Since $x_i(t)$ is ergodic, we may replace the average of measurements at different time t with the ensemble average. Starting from equation (M14), and since $W(\tau)$ is invariant, we may move the ensemble average bracket into the integral, as

$$\begin{aligned}
\langle A(u)\rangle_t &= \int d\tau\, W(\tau) \langle \left| \sum_i e^{-ju\left(x_i(t) + \frac{\tau}{2}\right)} \frac{\sin\left(u\left(r_i - \frac{\tau}{2}\right)\right)}{u} \right|^2 \rangle_t \\
&= \int d\tau\, W(\tau) \langle \sum_{i,j} e^{-ju\left[\left(x_i(t) + \frac{\tau}{2}\right) - \left(x_j(t) + \frac{\tau}{2}\right)\right]} \frac{\sin\left(u\left(r_i - \frac{\tau}{2}\right)\right)}{u} \frac{\sin\left(u\left(r_j - \frac{\tau}{2}\right)\right)}{u} \rangle_t \\
&= \int d\tau\, W(\tau) \int p(r_1) p(r_2) \frac{\sin\left(u\left(r_1 - \frac{\tau}{2}\right)\right)}{u} \frac{\sin\left(u\left(r_2 - \frac{\tau}{2}\right)\right)}{u} dr_1 dr_2 \langle \sum_{i,j} e^{-ju\left(x_i(t) - x_j(t)\right)} \rangle_t
\end{aligned} \qquad (M15)$$

The third equal sign comes from the fact that r and x are independent. The radius r_i follows the probability distribution $p(r)$, which is invariant. The x_i's are randomly distributed in space, forming an ergodic process. Therefore, the ensemble average over x is equal to the spatial average:

$$\langle \sum_{i,j} e^{-ju\left(x_i(t) - x_j(t)\right)} \rangle_t = \frac{1}{D^2} \int_{-\frac{D}{2}}^{\frac{D}{2}} e^{-ju(x_1 - x_2)} dx_1 dx_2 = \frac{4 \sin^2 \frac{Du}{2}}{D^2 u^2}, \qquad (M16)$$

Where D is the laser spot diameter. The term $\frac{Du}{2} = \pi$ term determines the average speckle size, which is consistent with the textbook[2] result.

$$\int p(r_1)p(r_2)\frac{\sin\left(u\left(r_1-\frac{\tau}{2}\right)\right)}{u}\frac{\sin\left(u\left(r_2-\frac{\tau}{2}\right)\right)}{u}dr_1dr_2=\left|\int p(r)\frac{\sin\left(u\left(r-\frac{\tau}{2}\right)\right)}{u}dr\right|^2 \qquad (M17)$$

If we substitute equations (M16) and (M17) back to equation (M15), we obtain

$$\langle A(u)\rangle=\frac{4\sin^2\frac{Du}{2}}{D^2u^2}\int d\tau\, W(\tau)\left|\int p(r)\frac{\sin\left(u\left(r-\frac{\tau}{2}\right)\right)}{u}dr\right|^2 \qquad (M18)$$

The particle size of our sample varies from ~50μm to ~1000μm, which is much larger than the wavelength 532nm. Thus, we meet the criterion $\lambda \ll H(x)$, implying that the phase correlation length is much smaller than the particle size. This is validated from our simulation in Fig. S5, so we may adopt the assumption $W(\tau)=\delta(\tau)$ safely. Therefore, we obtain the final expression (the same as equation 1),

$$\langle A(u)\rangle=\frac{4\sin^2\left(\frac{Du}{2}\right)}{D^2u^2}\left|\int p(r)\frac{\sin(ru)}{u}dr\right|^2. \qquad (M19)$$

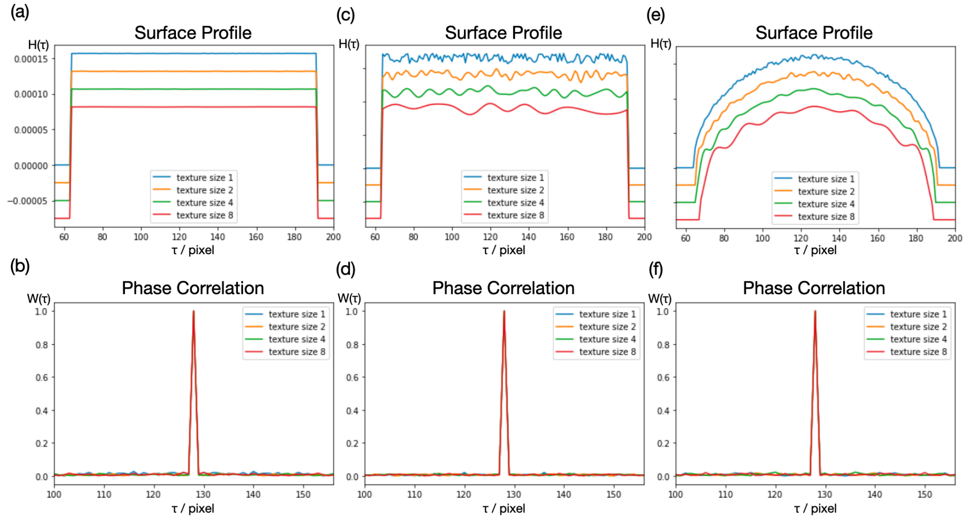

Fig. S5 | Simulation of phase correlation for different surface height profiles. (a) Surface profile for a square shape particle with a size ~150μm. The particle is sampled with 128 pixels. The height fluctuation is 1%, which corresponds to 1.5μm. Four curves with different texture sizes are plotted. The texture size describes the horizontal size of the fluctuations in pixel units. **(b)** plots the phase correlation calculated by $W(\tau)=\exp\left(j\frac{2\pi}{\lambda}H(\tau)\right)$ from the surface profile (a). **(c)** Surface profile with 10% height fluctuation. The rest information is the same with (a). **(d)** Phase correlation corresponding to (c). **(e)** Surface profile for a round particle with 5% height fluctuation. **(f)** The phase correlation corresponding to (e). The correlation lengths for all different height fluctuations and horizontal textures are smaller than a single pixel. Thus, we may safely assume that $W(\tau)\approx\delta(\tau)$.

4. Physics Enhanced AutoCorrelation-based Estimator (PEACE) algorithm

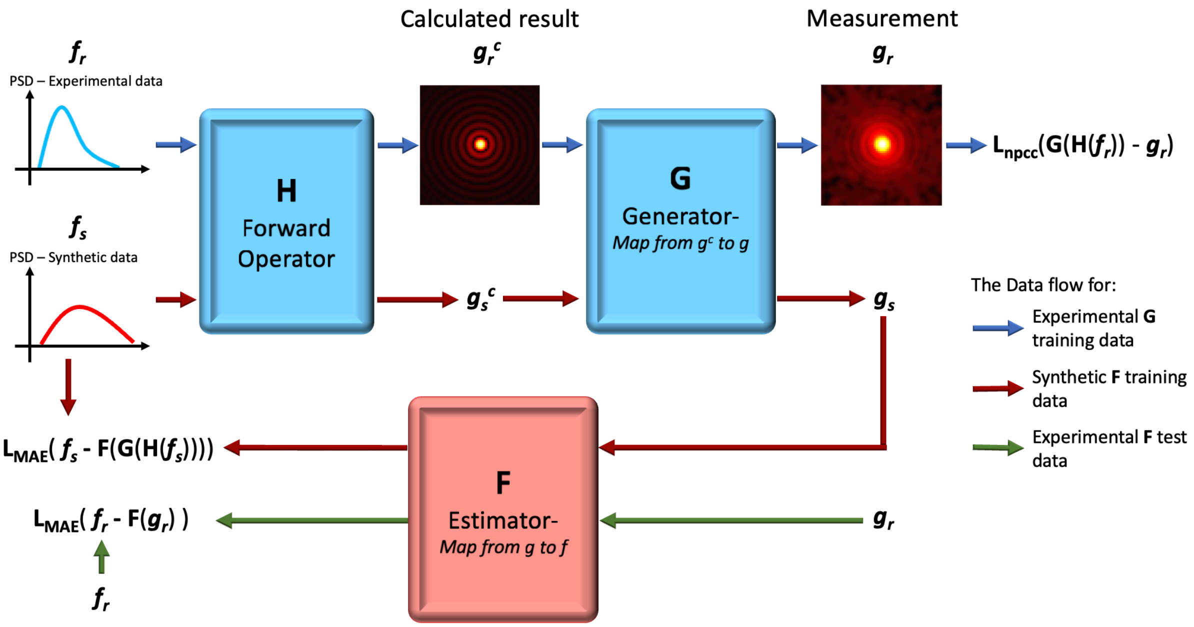

Fig. S6 | The learning scheme of the PEACE algorithm. A total of three data flows are described. First, an experimental dataset is utilized for training the generator with the NPCC loss function. Then a synthetic dataset is generated from the forward operator and the generator to train the estimator with the MAE loss. Finally, another experimental data serves as the test set for the estimator.

PEACE learning scheme. The forward relationship (1) is highly nonlinear; moreover, the analysis in Fig. 2 reveals that the inverse problem is also ill-conditioned: the relevant information for the PSD is in the sidelobes of the term $\frac{4\sin^2\left(\frac{uD}{2}\right)}{D^2u^2}$ and, hence, the solution may be disproportionately susceptible to detection noise and other disturbances. As a general strategy to stabilize the solution against disturbances, we must employ some form of regularization. In typical situations, the most effective regularizing priors are derived from sparsity arguments (also referred to as compressed sensing[3]). For linear inverse problems, sparse formulations lead to convex functionals that are numerically dealt with using methods such as TwIST and ADMM[4,5].

Our inverse problem with equation (1) is somewhat atypical, and not only because it is nonlinear. The probability for a specific radius $p(r)$ is manifestly modulated by $\frac{\sin(ru)}{u}$. The term $\frac{\sin(ru)}{u}$ approaches 0 as r approaches 0, adding to the ill-posedness already imposed by the preceding term $\frac{4\sin^2\left(\frac{uD}{2}\right)}{D^2u^2}$ term. This suggests that sparsifying the PSD as, for example, a superposition of radial basis functions, might be risky. Instead, we adopt a machine learning approach, using data to learn the regularizing prior. Since we can only

provide finite sample sets to get data from our experiment and the independent particle size analyzer, it is not enough to train a large neural network acting in "estimator" capacity to solve the inverse problem independently. Instead, with the help of the forward model, it is sufficient to complement the forward operator by training a small neural network called a "generator." Then we can generate a large synthetic dataset from the forward process to meet the data requirement of the training process for the estimator. The final answer is to solve the forward and inverse problems collaboratively. This algorithm is named "the Physics Enhanced AutoCorrelation-based Estimator (PEACE)."

Fig. S6 shows the PEACE learning scheme. It consists of three parts, forward operator $\mathbf{H}$, generator $\mathbf{G}$, and estimator $\mathbf{F}$. The forward operator $\mathbf{H}$ refers to equation 1, which produces the calculated result g^c from a given object f, the PSD in our case. The generator $\mathbf{G}$ maps g^c to the experimental measurement g. The estimator F is our ultimate final goal, which converts g to the corresponding object f. A paired small training dataset ($g_r = \mathbf{H}(f_r)$ and g_r^c) is collected from the experiment to train the generator $\mathbf{G}$ with the negative Pearson correlation coefficient (NPCC)[6,7] loss function. The generator $\mathbf{G}$ is designed based on the physical model to reach high performance with only 2.8k parameters, thus avoiding overfitting. A large synthetic dataset (g_s^c and f_s) is generated from $\mathbf{H}$ and $\mathbf{G}$ to train the estimator $\mathbf{F}$ with the mean-absolute-error (MAE) loss. A separated experimental dataset tests the performance of the estimator $\mathbf{F}$ on the real data.

The structure of the generator G. The generator contains 2.8k parameters. The generator compensates for the particles overlapping along the longitudinal direction and the deviation induced by the finite spatial integral and finite frames average in the ensemble autocorrelation calculation. It is designed based on the physical model. Random noise generated from a uniform distribution over $[0,1)$ is concatenated to the input to mimic the fluctuation induced by the effect of finite averaging. Moreover, adding noise can facilitate the generalization to the measured style since in the experimental measurement noise is inevitably present. Our forward operator only considers the top layer particles. The generator handles the influence of the underlying layers. Light scattered from the second layer particles will partially fill the gap between the first layer particles, resulting in a larger "effective particle size". Since the second layer particles are also densely located in the plane, this "large effective size" should be comparable to the beam spot size (5mm), which is much larger than the typical particle size (100 μm-1000 μm), resulting in a substantial intensity reduction of the high-order lobes in the experiment compared to the calculation result only from the first

layer. We assume that the multilayer effect is a blurring and non-linear function applied to the original calculation. This physical model can be written in the following expression,

$$g = \text{normalize}(\ g^c + h(g^c)\ ) \qquad (M20)$$

where $h(\cdot)$ denotes the nonlinear and convolutional blur operation. With this inspiration, we design the structure of **G** in the following way,

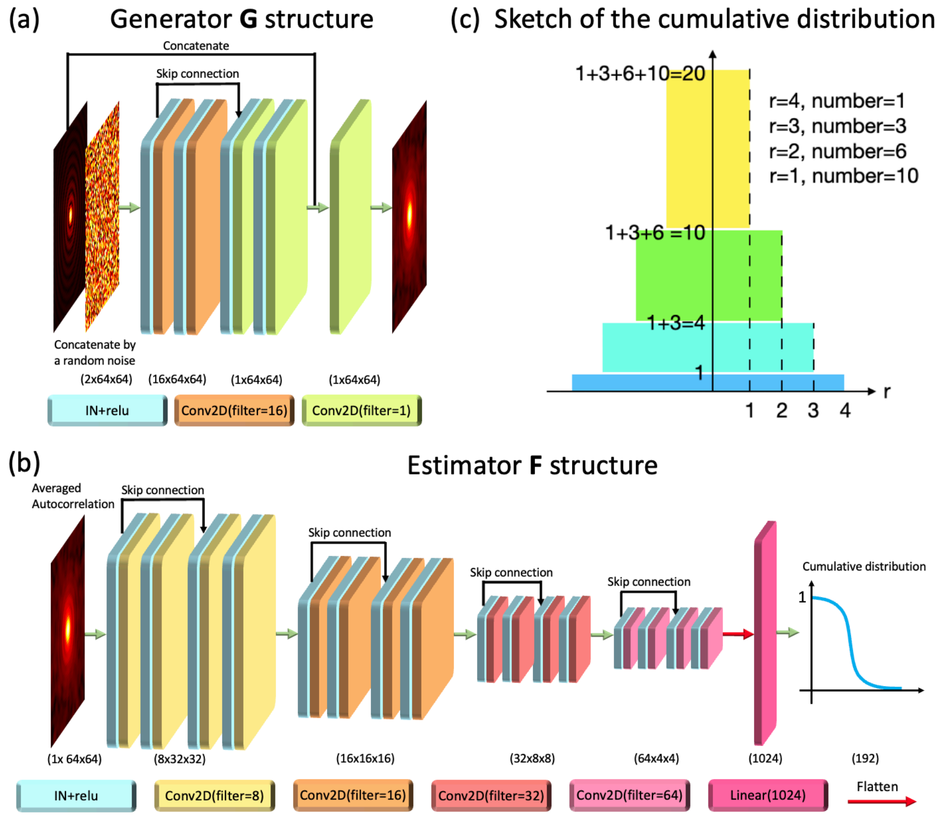

Fig. S7 | (a) Generator structure. The generator transforms the calculated image to the measured image. Random noise is generated and concatenated to the input. This neural network contains four convolutional layers. Instance-normalization (IN) and ReLU activation are applied before each convolutional layer except the last one. The last layer has a Sigmoid activation function. **(b) Estimator structure.** The estimator takes averaged autocorrelation images as input and the cumulative distributions as output. It consists of four stages with four convolutional layers in each. IN and ReLU activation are applied before each convolutional layer. There is a flattening layer and a linear layer with Sigmoid activation connected to the output of the fourth stage. The output data dimension and the filter parameters for each layer are labeled at the bottom of this figure. **(c)** This image is to show the physical meaning of the cumulative distribution. Assume there are 20 particles, ten particles have radius 1, six particles have radius 2, three particles have radius 3, and one particle has radius 4. If we stack them at the center and set the y-axis as the particle number, after normalization we find that the vertical "projections" are identical to the cumulative distribution.

$$g = \text{sigmoid}(\, g^c + \alpha \text{NN}(g^c) + \beta) \qquad (M21)$$

where the slope and bias constants α, β and the sigmoid function play the role of the last convolution layer with filter 1 and kernel size 1×1. The sigmoid function can scale the final output to $[0,1]$. The function $\text{NN}(\cdot)$ consists of four cascaded convolutional layers with 3×3 kernel and ReLU activation. It learns from data to fit $h(\cdot)$ without any downsampling. The detailed structure of **G** is shown in Fig. S7a. With this physics-inspired design, the generator **G** can reach high performance with only 2.8k parameters. This is a critical point to avoid overfitting with the small experimental training set.

The structure of the estimator F. The estimator contains 377k parameters. The structure of the estimator **F** is shown in detail in Fig. S7b. The ensemble averaged autocorrelation serves as the input. The estimator consists of four convolutional stages followed by a linear layer. Each stage has four 2D convolutional layers of kernel size 3, combined with Instance-Normalization layer (IN)[8] and ReLU activation. At each stage, the first convolution layer downsamples the image by a factor of 2. The filter depth increases gradually in each stage from 16 to 64. The skip connection structure serves to reduce the gradient vanishing effect[9]. After three convolution stages, the 3D signal is flattened and passed through a linear layer to the output, a non-parametric curve described by 192 samples. This is the cumulative distribution of the particle sizes, which we then differentiate to obtain the PSD. To reduce fluctuations, we bin the pixels of the PSD curve from 192 to 64 pixels.

There are two reasons why we pick up the cumulative distribution rather than the PSD directly as the output of the neural network. First, the cumulative distribution is monotonic from 0 to 1, which discourages overfitting the fluctuations that would inevitably appear in the PSD. Secondly, the cumulative distribution may easily be derived from Equation (1) as

$$\text{FT}\left(\int p(r)\frac{\sin(ru)}{u}dr\right)(x) = \int p(r)\,\text{Rect}\left(\frac{x}{r}\right)dr \; = \int_0^{|x|} p(r)dr \qquad (M22)$$

where $\text{Rect}(x) = 1$ when $x \in [-1,1]$, otherwise $= 0$ is the boxcar function. The physical meaning of the right-hand term in (M22) is shown in Fig. S7c. In effect, it moves all particles to the center, and the vertical axis becomes the number of particles as a function of r. After normalization, it becomes exactly the cumulative distribution. Moreover, this physical meaning applies to any particle shape besides round particles, and it is easily generalized to the rotationally averaged cumulative size distributions for non-rotationally symmetric cases, such as the cubic-shaped KCl powder particles shown in Fig.2a.

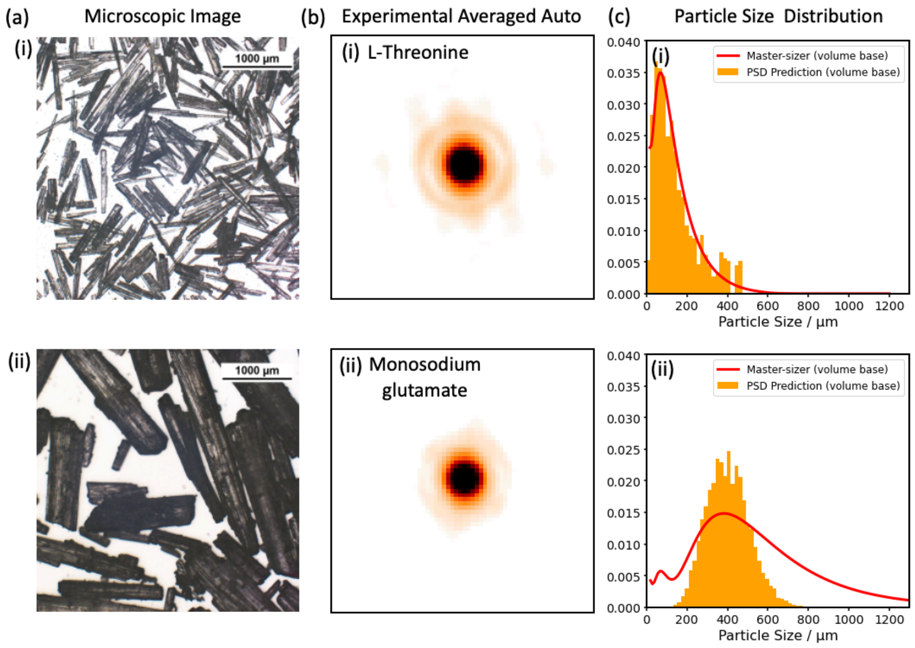

Fig. S8 | Validation results for the needle shape powders L-threonine (i) and Monosodium glutamate, MSG (ii). (a) In the microscopic images with the same scale bar for both (i) and (ii), MSG is clearly much bigger than L-threonine. **(b)** Measured averaged autocorrelations showing that MSG results in weaker side lobes compared to L-threonine. **(c)** Particle size distributions in volume base measured by our method (orange bar) and the Mastersizer (red line) serving as the ground truth.

To validate the performance for needle shape particles, we trained the pipeline with speckles from sieved KCl particles and tested it with L-threonine and Monosodium glutamate (MSG). The result is shown in Fig. S8. The microscope images in (a) show a visually clear size difference between these two powders, and verify that they are all needle-shaped. Part (b) shows averaged autocorrelations. According to. Fig. 2g in the main text, the first order side lobe is merged into the main lobe and is hard to resolve. L-threonine has a stronger second order side lobe than MSG. Higher order side lobes cannot be observed for either material. The particle size distributions transformed into volume base are plotted in part (c). The L-threonine's speckle prediction matches the Mastersizer result. The prediction for MSG has the same peak position as the ground truth but different widths. We may explain this width mismatch from two viewpoints. The first one is that the ground truth size distribution disperses more than 1000 µm (1 mm), which is out of the range in our training sets because KCl never forms particles as large. The second reason is that our model cannot work with the bimodal distribution very well, as we mention in the Discussion section of the main test and

further discuss in Supplementary Section 6. This experiment confirms that, to a certain extent, our model can apply to different particle shapes without retraining the neural network.

Details of the training process. The learning rate for the generator **G** is fixed to be 10^{-2} to avoid overfitting. The batch size is set to be 4. The training loss function is the negative Pearson correlation coefficient (NPCC)[6,7]. If k denotes the index in the training batch, and (i,j) denotes the $(i,j)^{th}$ th pixel in a particular image, then the batch-wise NPCC loss is defined as:

$$L_{\mathrm{NPCC}} = \sum_k \mathcal{E}_{\mathrm{NPCC}}(f_k, \hat{f}_k)$$

$$\mathcal{E}_{\mathrm{NPCC}}(f_k, \hat{f}_k) = -\frac{\sum_{i,j}\left(f_k(i,j) - \bar{f}_k\right)\left(\hat{f}_k(i,j) - \bar{\hat{f}}_k\right)}{\sqrt{\sum_{i,j}\left(f_k(i,j) - \bar{f}_k\right)^2}\sqrt{\sum_{i,j}\left(\hat{f}_k(i,j) - \bar{\hat{f}}_k\right)^2}} \qquad (M23)$$

Here, $\bar{\cdot}$ denotes spatial averaging. The ideal minimum value of this loss function is -1. Our validation NPCC loss can reach -0.995 after 50 epochs of training.

The learning rate of the estimator **F** was set to be 2×10^{-4} initially and halved whenever validation loss plateaued for 6 consecutive epochs. Batch size was set to 4. The training lasted for 100 epochs with mean absolute error (MAE) as the training loss function. For a 1D probability distribution, MAE is equivalent to the 1-Wasserstein distance[10]. The validation MAE loss for the synthetic data can reach 0.017 while it is 0.027 for the experimental validation data. This small mismatch is within our tolerance.

The computer used for training has Intel Xeon G6 CPU, 128 GB RAM, and dual Volta GPUs with 64 GB VRAM. Both training and test data were shuffled before each epoch. It took around 2 minutes to finish the whole training process.

5. Estimator results for the G-training dataset.

Fig. S8 plots the results for the dataset used to train the generator G. These data were disjoint from the training process of **F**. This plot is to cross-validate that **G** is not overfitting. If there is an overfitting effect, the performance of this dataset should be better than the test dataset. Compared to Fig.3, these two datasets have a similar performance from the second and the fifth columns, which indicates a well-trained generator.

From the first line result (50 μm – 106 μm) in this figure, we want to address that the prediction at the small size end has more deviation than the big size end. This phenomenon can also be interpreted by Equation (1), because the basis $\frac{\sin(ru)}{u}$ approaches 0 as r approaches 0, making its weight harder to distinguish at small radii. Moreover, we can reach a smaller r by modifying our optical system to generalize the interested size range in other applications. Based on the relationship $u = \frac{u'}{\lambda f_3}$, we can reduce the focal length of L3 f_3 to keep ru at the same value but with a smaller r and a larger u. In this way, we can push the lower bound even lower at the cost of compromising accuracy in the big size end of the predictions.

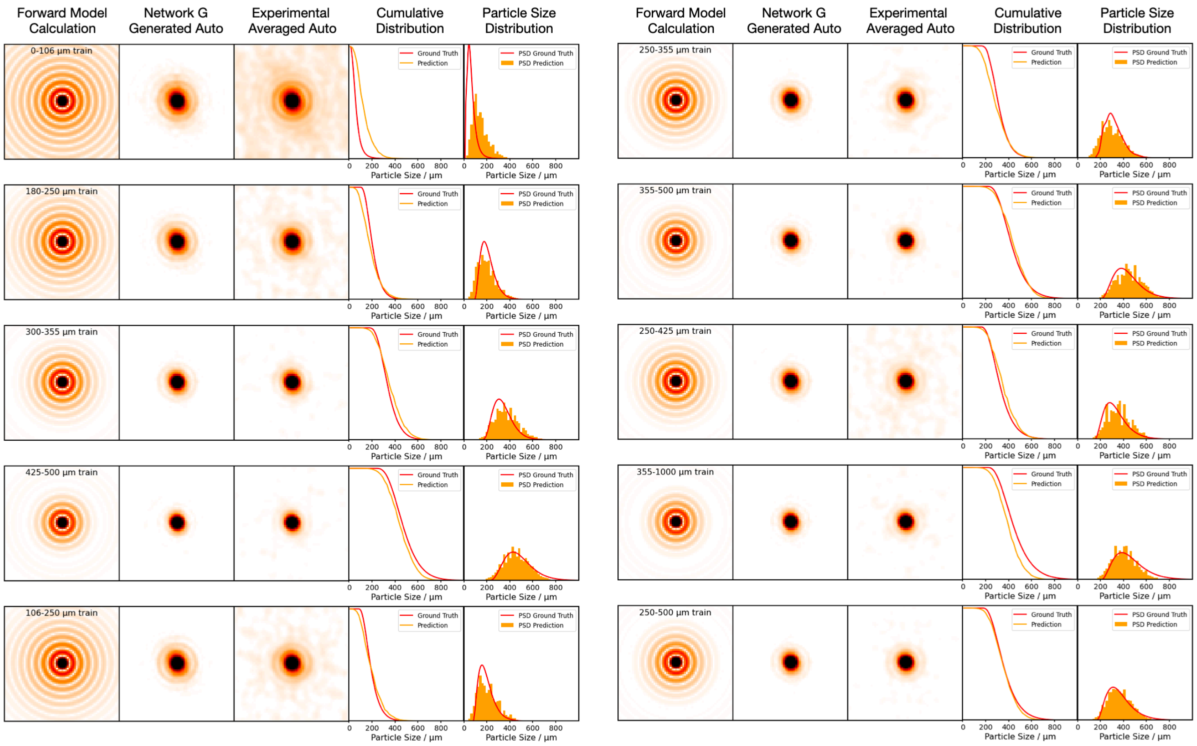

Fig. S9 | Prediction results by the generator and estimator with the generator G training dataset. Results of ten **G** training sample sets are plotted. These data were disjoint from the training process of the estimator **F**. The first and second columns show the output from the forward operator **H** and the generator **G**, respectively. The measured averaged autocorrelations are plotted in the third column. The prediction (marked as orange) from the estimator **F** and ground truth (marked as red) of the cumulative distributions and the corresponding PSDs are plotted together in columns 4 and 5.

6. Estimator stress test with a double peak PSD synthetic dataset.

We created a synthetic dataset with double-peak PSDs to test the estimator's generalization ability to different PSD shapes. Unfortunately, it fails to predict the double peaks PSDs, as shown in Fig. S10. The

output is a single peak PSD located between the two peaks. The position is approximately influenced by the weight of these two peaks. This is partially because this double peak feature is not included in the training dataset, so it has not been learned as a prior to the PEACE pipeline. However, even if we train the estimator with a mixture of single and double-peak PSD datasets, it cannot provide a good estimation. We can explain this with the forward model. To resolve a single peak in the PSD, the estimator could combine the low-order lobes' intensity with the learned prior from the dataset to estimate. However, high-order lobes are required to distinguish double peaks in the PSD. However, the higher-order lobes will merge into the background fluctuations when the large particles exist, as shown in Fig. 2e and the second column in Fig. S10. We may be able to solve this issue by increasing the number of averaging frames to suppress the background fluctuation feature in the autocorrelation image, or by exploiting higher order correlations (cumulants). This problem is outside the scope of our present work, but interesting as a topic for future research.

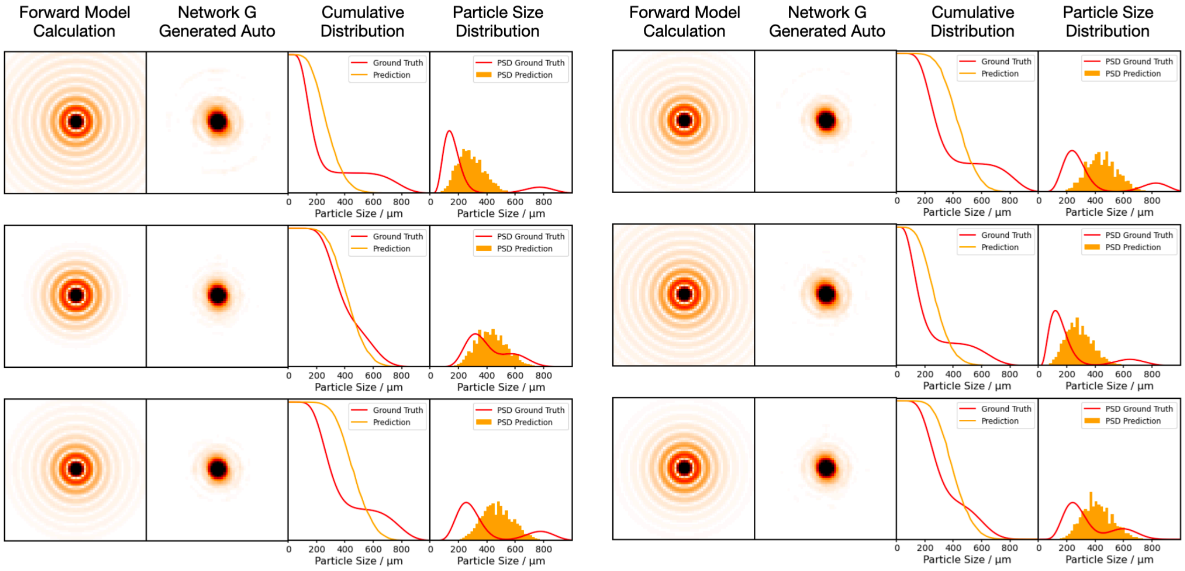

Fig. S10 | The stress test results with a double peak PSD synthetic dataset. The estimator fails to predict a double peak PSD. Instead, the estimation is a single peak PSD with the peak located between the two peaks.

References

1. Capellades, G. *et al.* A Compact Device for the Integrated Filtration, Drying, and Mechanical Processing of Active Pharmaceutical Ingredients. *J Pharm Sci* **109**, 1365–1372 (2020).

2. Dainty, J. C. *Laser speckle and related phenomena*. vol. 9 (Springer science & business Media, 2013).

3. Donoho, D. L. Compressed sensing. *IEEE Trans Inf Theory* **52**, 1289–1306 (2006).

4. Wei, E. & Ozdaglar, A. Distributed Alternating Direction Method of Multipliers. in *2012 IEEE 51st IEEE Conference on Decision and Control (CDC)* 5445–5450 (2012). doi:10.1109/CDC.2012.6425904.

5. Bioucas-Dias, J. M. & Figueiredo, Má. A. T. A New TwIST: Two-Step Iterative Shrinkage/Thresholding Algorithms for Image Restoration. *IEEE Transactions on Image Processing* **16**, 2992–3004 (2007).

6. Deng, M., Li, S., Goy, A., Kang, I. & Barbastathis, G. Learning to synthesize: robust phase retrieval at low photon counts. *Light Sci Appl* **9**, 36 (2020).

7. Li, S. & Barbastathis, G. Spectral pre-modulation of training examples enhances the spatial resolution of the phase extraction neural network (PhENN). *Opt. Express* **26**, 29340–29352 (2018).

8. Ulyanov, D., Vedaldi, A. & Lempitsky, V. S. Instance Normalization: The Missing Ingredient for Fast Stylization. *ArXiv* **abs/1607.0**, (2016).

9. He, K., Zhang, X., Ren, S. & Sun, J. Deep Residual Learning for Image Recognition. in *2016 IEEE Conference on Computer Vision and Pattern Recognition (CVPR)* 770–778 (2016). doi:10.1109/CVPR.2016.90.

10. Kolouri, S., Pope, P. E., Martin, C. E. & Rohde, G. K. Sliced-wasserstein autoencoder: An embarrassingly simple generative model. *arXiv preprint arXiv:1804.01947* (2018).
